# Lateralizing value of ictal head turning: A systematic review and meta‐analysis

**DOI:** 10.1002/epd2.70046

**Published:** 2025-05-23

**Authors:** Melita Cacic Hribljan, Georg Zimmermann, Sándor Beniczky

**Affiliations:** ^1^ Department of Clinical Neurophysiology Copenhagen University Hospital Rigshospitalet Copenhagen Denmark; ^2^ Department of Pediatrics Children's Hospital Srebrnjak Zagreb Croatia; ^3^ Department of Neurology, Neurological Intensive Care and Neurorehabilitation Christian Doppler University Hospital, Paracelsus Medical University and Center for Cognitive Neuroscience, Member of the European Reference Network EpiCARE Salzburg Austria; ^4^ Research Programme Biomedical Data Science Paracelsus Medical University Salzburg Austria; ^5^ Department of Artificial Intelligence and Human Interfaces Paris Lodron University Salzburg Austria; ^6^ Department of Clinical Neurophysiology Danish Epilepsy Center Dianalund Denmark; ^7^ Department of Clinical Neurophysiology Aarhus University Hospital and Department of Clinical Medicine, Aarhus University Aarhus Denmark; ^8^ Member of the European Reference Network EpiCARE Salzburg Austria

**Keywords:** epilepsy surgery, gyratory, nonversive, seizure, versive

## Abstract

**Objective:**

To elucidate the lateralizing value of ictal head turning in focal epilepsy and provide guidance for interpreting ictal semiology within the framework of presurgical evaluation.

**Methods:**

We conducted a systematic review of ictal head turning. We included studies reporting ictal head turning (versive, nonversive, and gyration) captured in video‐EEG recordings during presurgical evaluation. We assessed potential selection and assessment bias and evaluated confidence in the epileptogenic zone based on reported comparators—including resection site and surgical outcomes, intracerebral EEG, and MRI findings. Studies were classified as high quality if potential bias was low and confidence in the epileptogenic zone was high.

**Results:**

Versive head turning is usually contralateral to the epileptogenic zone (high level of evidence). Nonversive head turning is most often ipsilateral to the epileptogenic zone in temporal lobe epilepsy (high level of evidence) and contralateral in occipital lobe epilepsy (moderate level of evidence). In frontal lobe epilepsy, the lateralization of nonversive head turning may vary based on sub‐lobar localization, but high‐level evidence is lacking. Gyratory seizures are likely contralateral when initiated by versive head turning and evolving into focal‐to‐bilateral tonic–clonic seizures; in other cases, they may be ipsilateral, particularly in temporal and mesial frontal epilepsy, but the available evidence is insufficient for a definitive conclusion.

**Significance:**

The lateralizing value of ictal head turning depends on its specific characteristics and clinical context.

**Key points**

- Forced head version is usually contralateral to the focus, especially if followed by evolution to bilateral tonic‐clonic seizure.
- The lateralizing value of non‐forced head deviation depends on the context of the other data.
- In seizures from the temporal lobe, early non‐versive head deviation is usually ipsilateral.
- In seizures from the occipital lobe, non‐versive head deviation is contralateral.

## 1 INTRODUCTION

Head turning is often observed during seizures and has been used to lateralize the symptomatogenic zone, that is, to determine which hemisphere generates the symptoms.^1^ First described in stimulation studies conducted during surgery, head turning and eye deviation have traditionally been attributed to the activation of the contralateral frontal eye field and motor areas anterior to the precentral gyrus.^2^, ^3^ However, ictal head turning can present with various features, and its lateralizing significance may differ depending on the specific characteristics observed.

In this study, we used the following definitions for ictal head turning:

- *Version* (synonyms: *versive head turning, forced head turning*): Forced, involuntary, tonic or clonic head and eye deviations resulting in a sustained, unnatural positioning of the head and eyes. Often, the chin becomes elevated, and the movement may progress into a focal‐to‐bilateral tonic–clonic seizure.
- *Nonversive head turning* (synonyms: *orientation, nonforced head turning*): Voluntary‐like head turning resembling a physiological movement, past the midline but not reaching the shoulder, without a forced character or visible nuchal or limb muscle contractions. It often occurs relatively early in the seizure.
- *Gyration* (synonyms: *rotatory, circling, volvular, whole‐body turning*): Rotation around the body axis during a seizure, with a minimum of 180 degrees of rotation.

To clarify the lateralizing significance of ictal head turning, we conducted a systematic review and meta‐analysis. Our primary objective was to provide a state‐of‐the‐art, evidence‐based perspective on the anatomo‐clinical correlations of ictal head turning in focal epilepsy, aiming to guide the interpretation of ictal semiology in the context of presurgical evaluation.

## 2 METHODS

We conducted a systematic review and report its results following the Preferred Reporting Items for Systematic Reviews and Meta‐Analyses (PRISMA) guidelines.^4^

The study was pre‐registered in the International Prospective Register of Systematic Reviews—PROSPERO (CRD42023477434; https://www.crd.york.ac.uk/prospero/).

Our diagnostic PICO framework was defined as follows:

- *Population*: Patients with focal epilepsy beyond the neonatal age.
- *Index Test*: Version, nonversive head turning, or gyration.
- *Comparator Tests*: Resection site and effect of the operation, intracerebral EEG, MRI.
- *Outcome*: Accuracy in lateralizing the epileptogenic zone.

### 2.1 Search method and eligibility criteria

We searched in PubMed and EMBASE using the following string: (seizure OR epilep*) AND ((((((version) OR (versive)) OR (deviation)) OR (turning)) OR (gyration)) OR (gyratory)). The date last searched was November 30th, 2023.

We selected papers reporting the lateralizing value of ictal head turning with patient‐level data, published in peer‐reviewed journals, without language restrictions. Authors MCH and SB screened titles, abstracts, and full‐text articles for eligibility criteria. Disagreements were resolved during the full‐text screening and data abstraction phases through consensus discussions. For version and nonversive head turning, we included studies reporting a series of at least 10 patients. No such limitation was applied to publications reporting gyration.

### 2.2 Data extraction and evaluation

For each included publication, we extracted the number of reported patients with ictal head turning, the type of head turning, the results of the comparator tests, and data relevant to anatomo‐clinical correlations. The risk of bias for each publication was assessed using a version of the Quality Assessment of Diagnostic Accuracy Studies (QUADAS‐2), specifically adapted for studies on seizure semiology.^5^

- *Selection bias*: Risk of selection bias was assessed at the publication level, for each included paper, using the following criteria:
  ⚬ Was a consecutive or random sample of patients enrolled? (Yes/No/Unclear)
  ⚬ Was a case–control design used? (Yes/No/Unclear)
  ⚬ Did the study avoid inappropriate exclusions? (Yes/No/Unclear)

Based on the answers to these questions, the risk of selection bias was rated as Low, High, or Unclear.

- *Assessment bias*: Risk of assessment bias was also evaluated at the publication level, for each included paper, based on whether seizure semiology was interpreted blinded to other clinical data. The *risk of assessment bias was rated as Low, High, or Unclear*.

### 2.3 Reliability of the reference standard

Reliability of the reference standard was assessed at the individual patient level. We determined the level of confidence in the reported epileptogenic zone based on a recently developed method, which considers data from MRI, intracerebral EEG, and postoperative outcomes.^6^

The method distinguishes four confidence levels for localization of the epileptogenic zone:

⚬ *Very high*: Patients with Engel class IA outcomes after at least 1 year of postoperative follow‐up.
⚬ *High*: A well‐delineated focal lesion suspected to represent at least a part of the epileptogenic zone, or a clearly defined seizure onset zone identified by intracranial EEG, or Engel class I (but not specified IA) after at least 1 year of postoperative follow‐up.
⚬ *Moderate*: MRI signs of hippocampal sclerosis are suspected to be at least a part of the epileptogenic zone.
⚬ *Low*: Normal or multilobar MRI findings, or a poorly delineated seizure onset zone based on intracranial EEG, or Engel class II–IV postoperative outcomes.
  If multiple criteria were available and indicated different confidence levels, the level associated with postoperative outcome took precedence over intracranial EEG and MRI findings. Similarly, intracranial EEG findings took precedence over MRI results.

Each study was evaluated independently by authors MC and SB. Discordances were resolved by consensus discussions.

### 2.4 Meta‐analysis and statistics

We evaluated the odds ratio for correct lateralization based on the following hypotheses:

- Versive head turning suggests a contralateral epileptogenic zone.
- Nonversive head turning suggests an ipsilateral epileptogenic zone.

For each type of ictal head turning, we analyzed all included papers. Additionally, we performed a separate subgroup analysis focusing on high‐quality papers, defined as those with low selection and assessment bias and very high or high confidence in the epileptogenic zone. If these analyses yielded equivocal results or if the included studies showed significant heterogeneity, we further subdivided the data based on the lobar localization of the epileptogenic zone.

For each group and subgroup of included studies, we assessed heterogeneity^7^ and we calculated the odds ratio for correct lateralization and generated forest plots^8^ using R software (R Foundation for Statistical Computing, Vienna, Austria). For gyration, we did not find sufficient published evidence to conduct a systematic review and meta‐analysis. Therefore, we reviewed the available data on gyration using a narrative approach.

### 2.5 Assessment of the overall summary of evidence

The summary of evidence was assessed along the GRADE system^9^ according to the following *levels of evidence*:

- *Very low*: The true effect is likely to be markedly different from the estimated effect.
- *Low*: The true effect may be markedly different from the estimated effect.
- *Moderate*: The true effect is probably close to the estimated effect.
- *High*: There is high confidence that the true effect is similar to the estimated effect.

## 3 RESULTS

### 3.1 Versive and nonversive ictal head turning

**FIGURE 1:**
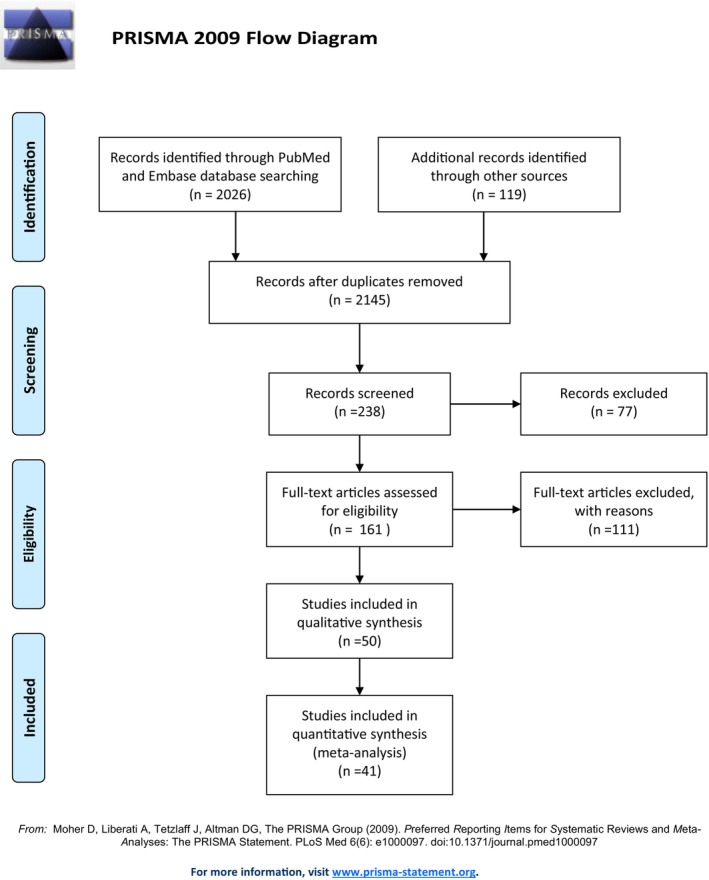
PRISMA flowchart for screening and selection of papers.

The PRISMA flow diagram (Figure 1) shows that out of the 2026 citations identified through the search strategy, 50 papers were included in the review, and 41 papers were included in the meta‐analyses, comprising a total of 857 patients. Tables 1 and 2 summarize the papers included in the review of versive and nonversive ictal head turning.

**TABLE 1:** The list of the papers included in the analysis of versive head turning.

Study	Author, year	Number of patients with the symptom (contralateral version) in region (all brain)	Total number of patients with symptom = version in region (all brain)	Risk of selection bias	Risk of assessment bias	Reliability of reference standard
1	Elwan, 2018^10^	14	14	Low	Low	Very high
2	Fotedar, 2022^11^	21	21	High	High	Undetermined
3	Wyllie, 1986^3^	61	61	Unclear	Low	Undetermined
4	Bleasel 1997^12^	20	20	Low	Low	Very high
5	Jayakar, 1992^13^	12	12	Low	Low	Very high
6	Chee, 1993^14^	17	17	Low	Low	Very high
7	Ochs, 1984^15^	19	43	Low	High	Undetermined
8	Kotagal, 1989^16^	8	8	Low	Low	Very high
9	Shukla, 2002^17^	6	7	Low	Low	High
10	Kotagal, 2000^18^	17	17	Low	Low	Very high
11	Marks, 1998^19^	15	15	Low	Low	Very high
12	Williamson, 1998^20^	25	25	Low	Low	Very high
13	Ataoğlu, 2015^21^	25	26	Low	Low	Very high
14	Wang, 2020^22^	45	58	Low	Low	Very high
15	Wang, 2013^23^	12	12	Low	Low	Very high
16	Duchowny, 1994^24^	10	10	Low	Low	High
17	Abarrategui, 2021^25^	13	13	Low	Low	Very high
18	Martinez‐Lizana, 2022^26^	16	25	Low	Low	High
19	Wyllie, 1986^27^	27	27	High	Low	Undetermined
20	Rémi, 2011^28^	45	45	High	High	High
21	Salanova, 1995^29^	5	5	Low	Low	Undetermined
22	Jobst, 2000^30^	11	12	Low	Low	High
23	Janszky, 2001^31^	6	6	Low	Low	Very high
24	Bonelli, 2007^32^	15	16	Low	Low	Undetermined
25	Chou, 2020^33^	3	4	Low	High	High
26	Morris, 1988^34^	2	2	Low	High	High
27	Lee, 2008^35^	14	15	Low	High	High
28	Harvey, 1993^36^	5	6	Low	High	Undetermined
29	Usui, 2011^37^	12	13	Low	High	High
30	Boesebeck, 2002^38^	14	14	Low	High	High
31	Olbrich, 2002^39^	4	4	Low	Low	Very high
32	Fernandez, 2018^40^	32	32	Low	Low	Undetermined
33	Rheims, 2005^41^	5	5	Low	High	High
34	Salanova, 1992^42^	4	4	Low	Low	High
35	van Dalen, 2024^43^	7	9	Low	High	High
36	Bartolomei, 2011^44^	4	11	Low	High	High
37	Yang, 2018^45^	4	4	Low	High	High

**TABLE 2:** The list of the papers included in the analysis of nonversive head turning.

Study	Author, year	Number of patients with the ipsilateral HD	Total number of patients with nonversive HD	Risk of selection bias	Risk of assessment bias	Reliability of reference standard
1	Wyllie, 1986^3^	7	13	Unclear	Low	Low
2	Chee, 1993^14^	8	10	Low	Low	Very high
3	Williamson, 1998^20^	33	34	Low	Low	Very high
4	Ataoğlu, 2015^21^	25	34	Low	Low	Very high
5	Abarrategui, 2021^25^	14	15	Low	Low	Very high
6	Nishimura, 2021^46^	8	11	Low	Low	Undetermined
7	Rémi, 2011^28^	35	35	High	High	Moderate
8	Jobst, 2000^30^	1	2	Low	Low	Very high
9	Janszky, 2001^31^	1	4	Low	Low	Very high
10	Bonelli, 2007^32^	2	5	Low	Low	Undetermined
11	Alqadi, 2016^47^	10	10	Low	Low	Very high
12	Salanova, 1992^42^	1	22	Low	Low	High
13	Rheims, 2005^41^	3	3	Low	High	High
14	van Dalen 2024^43^	13	17	Low	High	High
15	Liava, 2014^48^	0	4	Low	High	Very high

### 3.2 Versive head turning

**FIGURE 2:**
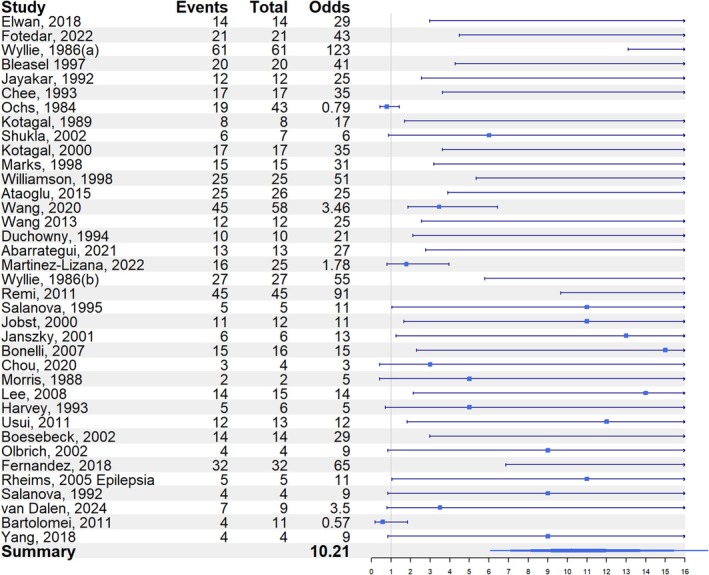
Odds ratio for contralateral localization of the epileptogenic zone relative to the versive head turning—all included studies.

**FIGURE 3:**
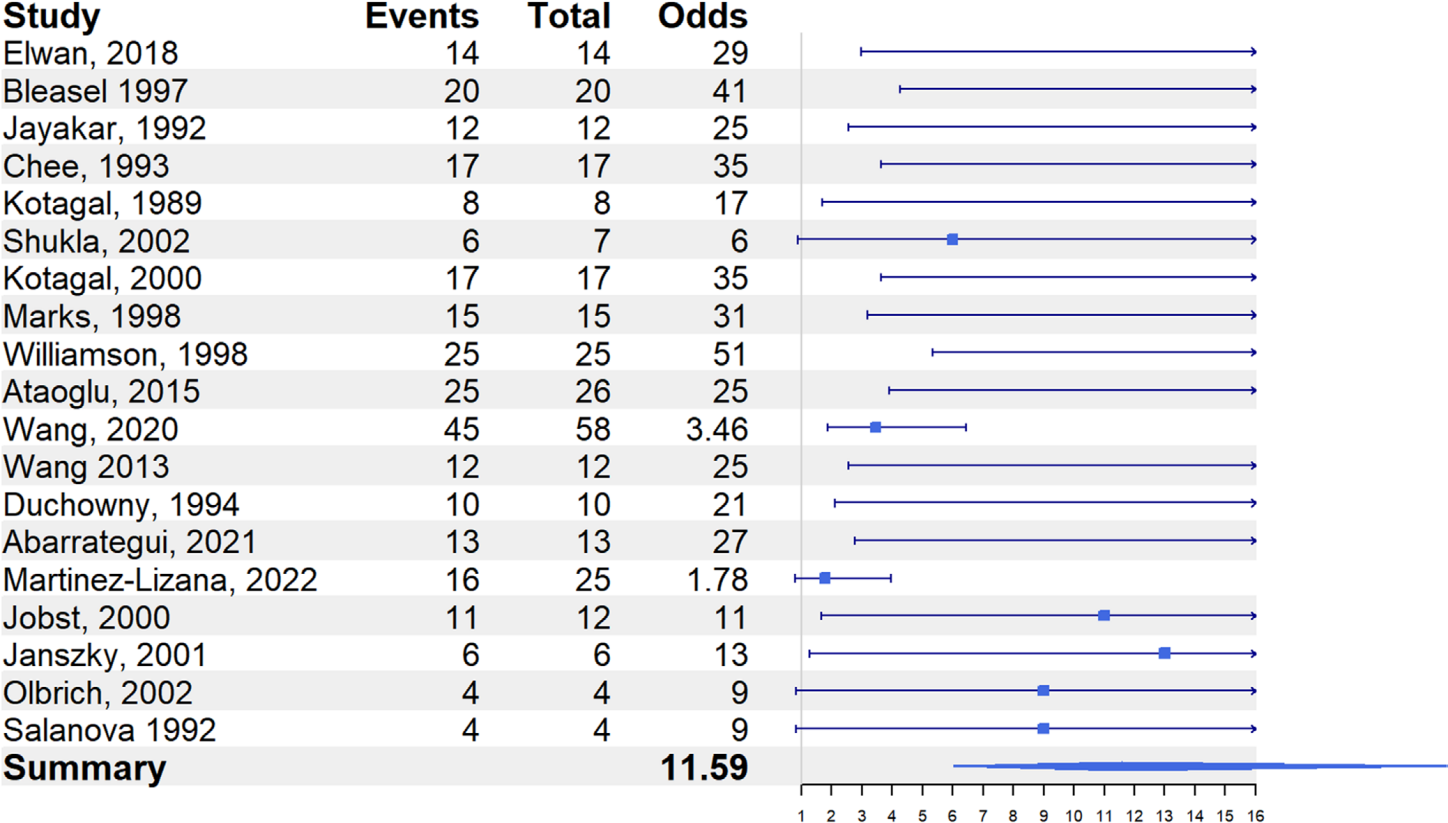
Odds ratio for contralateral localization of the epileptogenic zone relative to the versive head turning—high‐quality studies.

In total, 37 eligible papers were included in the analysis (Table 1 and Table S1), of which 19 were considered high‐quality and included in the subgroup analysis. Across all included papers, significant heterogeneity was observed (Test for heterogeneity: *p* < .0001). However, when restricting the analysis to the high‐quality papers, heterogeneity was no longer significant. Figures 2 and 3 present the forest plots for all included studies and the high‐quality studies, respectively. The odds ratio for contralateral localization of the epileptogenic zone was 10.2 (95% CI: 6.1–17.2) for all included studies and 11.6 (95% CI: 6.0–22.3) for the high‐quality studies.

### 3.3 Nonversive head turning

A total of 15 eligible papers were included in the analysis (Table 2 and Table S2), of which 11 were considered high‐quality and included in the subgroup analysis. Significant heterogeneity was observed across all included papers, and this remained significant when restricting the analysis to high‐quality papers (Test for heterogeneity: *p* < .0001). The odds ratio for ipsilateral localization of the epileptogenic zone was 2.4 across all papers and 2.2 in high‐quality papers (Figures S1 and S2). However, the lower limit of the 95% confidence interval (CI) was below 1 in both cases (0.9–6.0 across all papers; 0.7–7.4 in high‐quality papers), and hence revealed some uncertainty regarding the observed trend.

**FIGURE 4:**
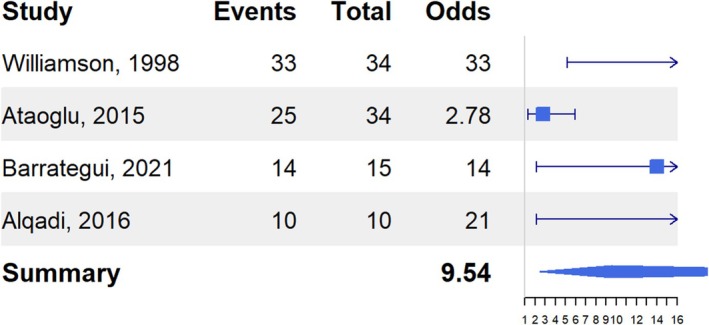
Odds ratio for ipsilateral localization of the epileptogenic zone relative to the nonversive head turning in temporal lobe epilepsy in high‐quality studies.

When the analysis was further restricted by grouping patients according to the lobar localization of the epileptogenic zone, heterogeneity was no longer significant for cases with the epileptogenic zone in the temporal lobe in high‐quality papers and in the occipital lobe in all papers. When the epileptogenic zone was located in the temporal lobe, the odds ratio for ipsilateral localization relative to nonversive head turning was 8.2 (95% CI: 2.2–30.8) across all papers and 9.5 (95% CI: 2.5–36.8) in high‐quality papers (Figure 4). In contrast, when the epileptogenic zone was in the occipital lobe, the odds ratio for ipsilateral localization was 0.1 (95% CI: 0.0–0.3). Since these values are below 1, the results indicate a contralateral localization of the epileptogenic zone. Reversing the hypothesis to test whether nonversive head turning in occipital cases indicates contralateral localization of the epileptogenic zone (Figure S3) yielded an odds ratio of 16.0 (95% CI: 3.1–83.7). However, this result was based on only two papers, with a total of 26 patients.

For cases with the epileptogenic zone in the frontal lobe, the odds ratio for ipsilateral localization was 2.5 across all studies and the same in high‐quality studies. However, the 95% CI crossed 1 in both cases (95% CI: 0.8–7.2 across all studies; 0.8–8.5 in high‐quality studies), indicating that nonversive head turning does not provide a clear lateralization value in frontal lobe epilepsy (Figures S4 and S5). Due to the limited number of cases, sub‐lobar analysis was not feasible.

### 3.4 Gyratory seizures

Gyratory seizures have been reported in focal epilepsies, primarily in the frontal and temporal lobes,^49^ as well as in generalized epilepsies, notably idiopathic generalized epilepsy (IGE)^50^, ^51^, ^52^, ^53^ Despite their occurrence, this semiology feature has been rarely investigated, and existing studies use varying definitions regarding the degree of rotation (a minimum of 90° or 180°), duration (a minimum of 3 or 10 s), and the involvement of different body parts. The highest number of rotations reported in the literature is 7.5.^54^ Once established, the direction of movement remains consistent in a given patient. Gyratory seizures may begin with a versive head movement in the same direction and can culminate in a focal‐to‐bilateral tonic–clonic (FBTC) seizure.

A study of 12 patients experiencing gyratory movements in 17 seizures^54^ suggested that the lateralizing value of these seizures depended on their evolution. Of the 12 patients, eight had frontal lobe epilepsy (FLE) and four had temporal lobe epilepsy (TLE), with half being nonlesional. During focal seizures, the direction of rotation lateralized the seizure onset zone to the contralateral hemisphere when head version initiated the gyration—observed in 58% of cases. Evolution into focal‐to‐bilateral tonic–clonic seizures occurred in 76% of gyratory seizures. When gyratory seizures were not preceded by head version, rotation tended to be ipsilateral to the seizure onset. Notably, all patients experienced amnesia for their rotatory movements.

In a cohort of 17 patients with hypermotor seizures who achieved seizure freedom following resective epilepsy surgery,^47^ 10 exhibited rotatory movements, with ipsilateral head and body turning ranging from 90° to 270°. Less than half (40%) had an epileptogenic zone in the frontal lobe. The authors concluded that the key lateralizing feature of hypermotor seizures was nonversive turning of the head and body, which was consistently ipsilateral to seizure onset, occurring in both frontal and extrafrontal cases.

In contrast, Leung et al. found that ictal body turning, defined as truncal turning of ≥90° without any tonic element in the extremities, was localizing to the mesial frontal region but was equally likely to be ipsilateral or contralateral.^55^ Their study of 28 patients with 55 mesial frontal lobe seizures found that none of the patients exhibited concurrent versive head turning.

Shukla et al. investigated the lateralizing significance of unidirectional whole‐body turning in patients with complex partial seizures originating from the temporal lobe.^56^ Among 330 patients undergoing long‐term video‐EEG monitoring, 13 exhibited unidirectional whole‐body turning, defined as rotation of the trunk, head, and limbs by >90° lasting >10 s. In 11 of 13 patients (84.7%) and 26 of 28 seizures (92.8%), whole‐body turning was contralateral to the seizure onset zone.

Another study examining the role of ictal body turning in the lateralization of focal epilepsies included 263 patients.^57^ The authors categorized body turning into two groups: nonversive (at least 90° of nonforced rotation without tonic or clonic components) and versive (at least 90° of forced rotation with tonic or clonic components). Head turning was analyzed separately and further divided into nonversive and versive, making comparisons with previous studies difficult. Ictal body turning was observed in patients with both temporal lobe (19 patients) and extratemporal (8 patients) epilepsy. Nonversive body turning was seen only during complex partial seizures and was ipsilateral to the epileptogenic zone. Conversely, versive body turning occurred right before or during the tonic phase of FBTC seizures and was contralateral to the epileptogenic zone.

A recently published study analyzed seizure semiology in 50 pediatric patients who achieved seizure freedom after frontal lobe resective surgery.^43^ It found that 27.7% of patients exhibited ictal body turning, occurring either ipsilaterally or contralaterally. Among children with mesial seizure onset, ictal body turning was more frequent (55.6%) compared to those with lateral seizure onset (13%). Ipsilateral body turning was significantly associated with mesial frontal seizure onset. In this study, ictal body turning was defined as “truncal turning of 90° or more without any tonic element of the extremities, occurring parallel to the body axis and horizontally.”

## 4 DISCUSSION

The systematic review demonstrated that the lateralizing value of ictal head turning depends on its characteristics and the context in which it occurs. Versive ictal head turning is usually contralateral to the epileptogenic zone, regardless of lobar localization (high level of evidence).

Version as a contralaterally lateralizing sign is particularly reliable when observed immediately prior to the tonic–clonic phase of focal‐to‐bilateral tonic–clonic seizures (also known as secondary generalization), typically within 2–36 s (mean: 15 s), and is seen in both temporal and extratemporal cases.^3^, ^10^, ^14^, ^18^, ^19^, ^27^, ^30^ Head deviation that continues through the generalization phase is considered the most reliable lateralizing sign, identifying a contralateral seizure focus in over 90% of cases.^58^ When the contralateral version does not persist into generalization, a late ipsilateral version may occur at the end of the seizure,^14^, ^27^ possibly due to mechanisms similar to those underlying ipsilateral last clonic jerks. As with most aspects of seizure semiology, exceptions exist: in rare cases, version may be ipsilateral, especially when ictal activity rapidly propagates from one hemisphere to the other before generalization.^32^, ^59^, ^60^

Seizures often exhibit head deviations in different directions at various points during their progression. Typically, an initial nonforced head deviation—usually ipsilateral to the seizure focus (most often in temporal lobe cases)—is followed by a forced head deviation (version) directed contralaterally, just before secondary generalization. In seizures with rapid secondary generalization, the initial movement may instead be a forced contralateral head deviation.^58^

The lateralizing value of nonversive ictal head turning varies depending on the lobar localization of the epileptogenic zone. It is ipsilateral to the epileptogenic zone when localized in the temporal lobe (high level of evidence) and contralateral when localized in the occipital lobe (moderate level of evidence).

It is important to emphasize that while these findings are significant at a group level, notable exceptions exist. Therefore, interpretation at the individual patient level should consider all other available data during the presurgical evaluation. It is not recommended to take individual semiologic features as “proof” of localization or lateralization, but rather to think about the co‐occurrence and temporal evolution of all ictal symptoms and signs when trying to infer possible cerebral organization of seizures for an individual patient, in addition to all other available data.^61^ Another important limitation is that in multilobar cases, the confidence in the epileptogenic zone is low, and therefore it was not included in the meta‐analysis.

We were unable to unequivocally demonstrate the lateralizing value of nonversive ictal head turning in patients with frontal lobe epilepsy, although there was a trend toward ipsilateral lateralization (low level of evidence). This is likely due to variability in lateralization at the sub‐lobar level in frontal lobe epilepsy. However, high‐level evidence is lacking, as the number of published cases for each sub‐lobar region remains too low. High‐quality, multi‐center studies are needed to clarify this issue.

The heterogeneity of the studies on gyratory seizures did not allow meta‐analysis. Several studies suggest that gyration is contralateral to the epileptogenic zone when initiated by versive head turning and when it evolves into a focal‐to‐bilateral tonic–clonic seizure. In contrast, gyration without versive initiation or secondary generalization may be ipsilateral when the epileptogenic zone is located in the temporal lobe or mesial frontal structures (very low level of evidence).

## Supporting information


Figure S1.

Figure S2.

Figure S3.

Figure S4.

Figure S5.

Table S1.

Table S2.

Figure Caption.

## Data Availability

Data sharing is not applicable to this article as no new data were created or analyzed in this study.

## References

[1] Beniczky S , Tatum WO , Blumenfeld H , Stefan H , Mani J , Maillard L , et al. Seizure semiology: ILAE glossary of terms and their significance. Epileptic Disord. 2022;24(3):447–495. 35770761 10.1684/epd.2022.1430

[2] Godoy J , Lüders H , Dinner DS , Morris HH , Wyllie E . Versive eye movements elicited by cortical stimulation of the human brain. Neurology. 1990;40(2):296–299. 2300252 10.1212/wnl.40.2.296

[3] Wyllie E , Lüders H , Morris HH , Lesser RP , Dinner DS . The lateralizing significance of versive head and eye movements during epileptic seizures. Neurology. 1986;36(5):606–611. 3703259 10.1212/wnl.36.5.606

[4] Page MJ , McKenzie JE , Bossuyt PM , Boutron I , Hoffmann TC , Mulrow CD , et al. The PRISMA 2020 statement: an updated guideline for reporting systematic reviews. BMJ. 2021;372:n71. 10.1136/bmj.n71 33782057 PMC8005924

[5] Whiting PF , Rutjes AW , Westwood ME , Mallett S , Deeks JJ , Reitsma JB , et al. QUADAS‐2: a revised tool for the quality assessment of diagnostic accuracy studies. Ann Intern Med. 2011;155(8):529–536. 10.7326/0003-4819-155-8-201110180-00009 22007046

[6] Ryvlin P , Barba C , Bartolomei F , Baumgartner C , Brazdil M , Fabo D , et al. Grading system for assessing the confidence in the epileptogenic zone reported in published studies: a Delphi consensus study. Epilepsia. 2024;65(5):1346–1359. 38420750 10.1111/epi.17928

[7] Higgins JP , Thompson SG . Quantifying heterogeneity in a meta‐analysis. Stat Med. 2002;21(11):1539–1558. 10.1002/sim.1186 PMID: 12111919. 12111919

[8] Balduzzi S , Rücker G , Schwarzer G . How to perform a meta‐analysis with R: a practical tutorial. Evid Based Ment Health. 2019;22:153–160. 31563865 10.1136/ebmental-2019-300117 PMC10231495

[9] Guyatt GH , Oxman AD , Vist GE , Kunz R , Falck‐Ytter Y , Alonso‐Coello P , et al. GRADE: an emerging consensus on rating quality of evidence and strength of recommendations. BMJ. 2008;336(7650):924–926. 18436948 10.1136/bmj.39489.470347.AD PMC2335261

[10] Elwan S , Alexopoulos A , Silveira DC , Kotagal P . Lateralizing and localizing value of seizure semiology: comparison with scalp EEG, MRI and PET in patients successfully treated with resective epilepsy surgery. Seizure. 2018;61:203–208. 30216856 10.1016/j.seizure.2018.08.026

[11] Fotedar N , Gajera P , Pyatka N , Nasralla S , Kubota T , Vaca GF , et al. A descriptive study of eye and head movements in versive seizures. Seizure. 2022;98:44–50. 10.1016/j.seizure.2022.04.003 35417829

[12] Bleasel A , Kotagal P , Kankirawatana P , Rybicki L . Lateralizing value and semiology of ictal limb posturing and version in temporal lobe and extratemporal epilepsy. Epilepsia. 1997;38(2):168–174. 9048668 10.1111/j.1528-1157.1997.tb01093.x

[13] Jayakar P , Duchowny M , Resnick T , Alvarez L . Ictal head deviation: lateralizing significance of the pattern of head movement. Neurology. 1992;42(10):1989–1992. 1407581 10.1212/wnl.42.10.1989

[14] Chee MW , Kotagal P , Van Ness PC , Gragg L , Murphy D , Lüders HO . Lateralizing signs in intractable partial epilepsy: blinded multiple‐observer analysis. Neurology. 1993;43(12):2519–2525. 10.1212/wnl.43.12.2519 8255450

[15] Ochs R , Gloor P , Quesney F , Ives J , Olivier A . Does head‐turning during a seizure have lateralizing or localizing significance? Neurology. 1984;34(7):884–890. 6539865 10.1212/wnl.34.7.884

[16] Kotagal P , Lüders H , Morris HH , Dinner DS , Wyllie E , Godoy J , et al. Dystonic posturing in complex partial seizures of temporal lobe onset: a new lateralizing sign. Neurology. 1989;39(2 Pt 1):196–201. 2915789 10.1212/wnl.39.2.196

[17] Shukla G , Bhatia M , Gaekwad SB , Singh VP , Jain S , Maheshwari MC . The lateralizing significance of version of head and dystonic limb posturing in epileptic seizures. Neurol India. 2002;50(1):33–36. 11960148

[18] Kotagal P , Bleasel A , Geller E , Kankirawatana P , Moorjani BI , Rybicki L . Lateralizing value of asymmetric tonic limb posturing observed in secondarily generalized tonic‐clonic seizures. Epilepsia. 2000;41(4):457–462. 10756413 10.1111/j.1528-1157.2000.tb00189.x

[19] Marks WJ Jr , Laxer KD . Semiology of temporal lobe seizures: value in lateralizing the seizure focus. Epilepsia. 1998;39(7):721–726. 9670900 10.1111/j.1528-1157.1998.tb01157.x

[20] Williamson PD , Thadani VM , French JA , Darcey TM , Mattson RH , Spencer SS , et al. Medial temporal lobe epilepsy: videotape analysis of objective clinical seizure characteristics. Epilepsia. 1998;39(11):1182–1188. 9821982 10.1111/j.1528-1157.1998.tb01309.x

[21] Ataoğlu EE , Yıldırım İ , Bilir E . An evaluation of lateralizing signs in patients with temporal lobe epilepsy. Epilepsy Behav. 2015;47:115–119. 25989878 10.1016/j.yebeh.2015.04.015

[22] Wang Y , Wang X , Sang L , Zhang C , Zhao BT , Mo JJ , et al. Network of ictal head version in mesial temporal lobe epilepsy. Brain Behav. 2020;10(11):e01820. 32857475 10.1002/brb3.1820 PMC7667364

[23] Wang F , Liu X , Pan S , Wang M , Chen S . Electroclinical characteristics of posterior lateral temporal epilepsy. Epilepsy Behav. 2013;26(1):126–131. 23200534 10.1016/j.yebeh.2012.09.036

[24] Duchowny M , Jayakar P , Resnick T , Levin B , Alvarez L . Posterior temporal epilepsy: electroclinical features. Ann Neurol. 1994;35(4):427–431. 8154869 10.1002/ana.410350409

[25] Abarrategui B , Mai R , Sartori I , Francione S , Pelliccia V , Cossu M , et al. Temporal lobe epilepsy: a never‐ending story. Epilepsy Behav. 2021;122:108122. 34175663 10.1016/j.yebeh.2021.108122

[26] Martinez‐Lizana E , Brandt A , Foit NA , Urbach H , Schulze‐Bonhage A . Ictal semiology of epileptic seizures with insulo‐opercular genesis. J Neurol. 2022;269(6):3119–3128. 34812940 10.1007/s00415-021-10911-0 PMC9120119

[27] Wyllie E , Lüders H , Morris HH , Lesser RP , Dinner DS , Goldstick L . Ipsilateral forced head and eye turning at the end of the generalized tonic‐clonic phase of versive seizures. Neurology. 1986;36(9):1212–1217. 3748388 10.1212/wnl.36.9.1212

[28] Rémi J , Wagner P , O'Dwyer R , Silva Cunha JP , Vollmar C , Krotofil I , et al. Ictal head turning in frontal and temporal lobe epilepsy. Epilepsia. 2011;52(8):1447–1451. 10.1111/j.1528-1167.2011.03076.x 21627643

[29] Salanova V , Morris HH , Van Ness P , Kotagal P , Wyllie E , Lüders H . Frontal lobe seizures: electroclinical syndromes. Epilepsia. 1995;36(1):16–24. 8001503 10.1111/j.1528-1157.1995.tb01659.x

[30] Jobst BC , Siegel AM , Thadani VM , Roberts DW , Rhodes HC , Williamson PD . Intractable seizures of frontal lobe origin: clinical characteristics, localizing signs, and results of surgery. Epilepsia. 2000;41(9):1139–1152. 10999553 10.1111/j.1528-1157.2000.tb00319.x

[31] Janszky J , Fogarasi A , Jokeit H , Ebner A . Lateralizing value of unilateral motor and somatosensory manifestations in frontal lobe seizures. Epilepsy Res. 2001;43(2):125–133. 11164701 10.1016/s0920-1211(00)00186-8

[32] Bonelli SB , Lurger S , Zimprich F , Stogmann E , Assem‐Hilger E , Baumgartner C . Clinical seizure lateralization in frontal lobe epilepsy. Epilepsia. 2007;48(3):517–523. 17346249 10.1111/j.1528-1167.2006.00943.x

[33] Chou CC , Lee CC , Lin CF , Peng SJ , Hsiao FJ , Yu HY , et al. Cingulate gyrus epilepsy: semiology, invasive EEG, and surgical approaches. Neurosurg Focus. 2020;48(4):E8. 10.3171/2020.1.FOCUS19914 32234986

[34] Morris HH 3rd , Dinner DS , Lüders H , Wyllie E , Kramer R . Supplementary motor seizures: clinical and electroencephalographic findings. Neurology. 1988;38(7):1075–1082. 3386826 10.1212/wnl.38.7.1075

[35] Lee JJ , Lee SK , Lee SY , Park KI , Kim DW , Lee DS , et al. Frontal lobe epilepsy: clinical characteristics, surgical outcomes and diagnostic modalities. Seizure. 2008;17(6):514–523. 18329907 10.1016/j.seizure.2008.01.007

[36] Harvey AS , Hopkins IJ , Bowe JM , Cook DJ , Shield LK , Berkovic SF . Frontal lobe epilepsy: clinical seizure characteristics and localization with ictal 99mTc‐HMPAO SPECT. Neurology. 1993;43(10):1966–1980. 8413954 10.1212/wnl.43.10.1966

[37] Usui N , Mihara T , Baba K , Matsuda K , Tottori T , Umeoka S , et al. Versive seizures in occipital lobe epilepsy: lateralizing value and pathophysiology. Epilepsy Res. 2011;97(1–2):157–161. 21885252 10.1016/j.eplepsyres.2011.08.004

[38] Boesebeck F , Schulz R , May T , Ebner A . Lateralizing semiology predicts the seizure outcome after epilepsy surgery in the posterior cortex. Brain. 2002;125(Pt 10):2320–2331. 12244088 10.1093/brain/awf236

[39] Olbrich A , Urak L , Gröppel G , Serles W , Novak K , Porsche B , et al. Semiology of temporal lobe epilepsy in children and adolescents. Value in lateralizing the seizure onset zone [corrected] [published correction appears in epilepsy res 2002; 51(1‐2): 211.]. Epilepsy Res. 2002;48(1–2):103–110. 11823114 10.1016/s0920-1211(01)00326-6

[40] Fernandez‐Baca Vaca G , Mayor CL , Losarcos NG , Park JT , Lüders HO . Epileptic seizure semiology in different age groups. Epileptic Disord. 2018;20(3):179–188. 29905152 10.1684/epd.2018.0970

[41] Rheims S , Demarquay G , Isnard J , Guenot M , Fischer C , Sindou M , et al. Ipsilateral head deviation in frontal lobe seizures. Epilepsia. 2005;46(11):1750–1753. 16302854 10.1111/j.1528-1167.2005.00293.x

[42] Salanova V , Andermann F , Olivier A , Rasmussen T , Quesney LF . Occipital lobe epilepsy: electroclinical manifestations, electrocorticography, cortical stimulation and outcome in 42 patients treated between 1930 and 1991. Surgery of occipital lobe epilepsy. Brain. 1992;115(Pt 6):1655–1680. 1486456 10.1093/brain/115.6.1655

[43] van Dalen T , Kirkham JF , Chari A , D'Arco F , Moeller F , Eltze C , et al. Characterizing frontal lobe seizure semiology in children. Ann Neurol. 2024;95(6):1138–1148. 38624073 10.1002/ana.26922

[44] Bartolomei F , Gavaret M , Hewett R , Valton L , Aubert S , Régis J , et al. Neural networks underlying parietal lobe seizures: a quantified study from intracerebral recordings. Epilepsy Res. 2011;93(2–3):164–176. 21227653 10.1016/j.eplepsyres.2010.12.005

[45] Yang Y , Wang H , Zhou W , Qian T , Sun W , Zhao G . Electroclinical characteristics of seizures arising from the precuneus based on stereoelectroencephalography (SEEG). BMC Neurol. 2018;18(1):110. 30103717 10.1186/s12883-018-1119-z PMC6088396

[46] Nishimura M , Okanishi T , Itamura S , Homma Y , Sakakura K , Ichikawa N , et al. Seizure focus in the frontal interhemispheric fissure leads to ipsilateral isolated eye deviation. Epilepsy Behav. 2021;116:107772. 33556862 10.1016/j.yebeh.2021.107772

[47] Alqadi K , Sankaraneni R , Thome U , Kotagal P . Semiology of hypermotor (hyperkinetic) seizures. Epilepsy Behav. 2016;54:137–141. 26708064 10.1016/j.yebeh.2015.11.017

[48] Liava A , Mai R , Tassi L , Cossu M , Sartori I , Nobili L , et al. Paediatric epilepsy surgery in the posterior cortex: a study of 62 cases. Epileptic Disord. 2014;16(2):141–164. 10.1684/epd.2014.0648 24853765

[49] Saka E , Saygi S , Ciğer A , Selekler K . Circling seizures. Seizure. 1996;5(4):299–302. 8952016 10.1016/s1059-1311(96)80024-0

[50] Gastaut H , Aguglia U , Tinuper P . Benign versive or circling epilepsy with bilateral 3‐cps spike‐and‐wave discharges in late childhood. Ann Neurol. 1986;19(3):301–303. 3963776 10.1002/ana.410190316

[51] Aguglia U , Gambardella A , Le Piane E , Messina D , Russo C , Oliveri RL , et al. Idiopathic generalized epilepsies with versive or circling seizures. Acta Neurol Scand. 1999;99(4):219–224. 10.1111/j.1600-0404.1999.tb07350.x 10225351

[52] Ferrie CD . Idiopathic generalized epilepsies imitating focal epilepsies. Epilepsia. 2005;46(Suppl 9):91–95. 10.1111/j.1528-1167.2005.00319.x 16302881

[53] Ortiz‐Guerrero G , Perez‐Ortiz JM , Saify M , Selcen D , Fine AL . Gyratory seizures as a manifestation of possible generalized epilepsy. Epileptic Disord. 2024;26(5):718–720. 38984496 10.1002/epd2.20247

[54] Dobesberger J , Walser G , Embacher N , Unterberger I , Luef G , Bauer G , et al. Gyratory seizures revisited: a video‐EEG study. Neurology. 2005;64(11):1884–1887. 15955938 10.1212/01.WNL.0000163774.24004.8F

[55] Leung H , Schindler K , Clusmann H , Bien CG , Pöpel A , Schramm J , et al. Mesial frontal epilepsy and ictal body turning along the horizontal body axis. Arch Neurol. 2008;65(1):71–77. 18195141 10.1001/archneurol.2007.22

[56] Shukla G , Bhatia M , Padma Srivastava MV , Tripathi M , Srivastava A , Singh VP , et al. Unidirectional whole body turning: a new lateralising sign in complex partial seizures. J Neurol Neurosurg Psychiatry. 2005;76(12):1726–1729. 16291904 10.1136/jnnp.2004.042549 PMC1739458

[57] Mercan M , Yıldırım İ , Akdemir Ö , Bilir E . Ictal body turning in focal epilepsy. Epilepsy Behav. 2015;44:253–257. 25769674 10.1016/j.yebeh.2014.11.005

[58] Kernan JC , Devinsky O , Luciano DJ , Vazquez B , Perrine K . Lateralizing significance of head and eye deviation in secondary generalized tonic‐clonic seizures. Neurology. 1993;43(7):1308–1310. 8327129 10.1212/wnl.43.7.1308

[59] Fakhoury T , Abou‐Khalil B , Peguero E . Differentiating clinical features of right and left temporal lobe seizures. Epilepsia. 1994;35(5):1038–1044. 7925149 10.1111/j.1528-1157.1994.tb02552.x

[60] Marashly A , Ewida A , Agarwal R , Younes K , Lüders HO . Ictal motor sequences: lateralization and localization values. Epilepsia. 2016;57(3):369–375. 26864781 10.1111/epi.13322

[61] McGonigal A , Bartolomei F , Chauvel P . On seizure semiology. Epilepsia. 2021;62(9):2019–2035. 10.1111/epi.16994 34247399

