# Supplementary figures and images for: Lateralizing value of ictal head turning: A systematic review and meta‐analysis
Source: Epileptic Disord. 2025 May 23;27(4):568–78. doi: 10.1002/epd2.70046 (PMC12398197; doi:10.1002/epd2.70046)

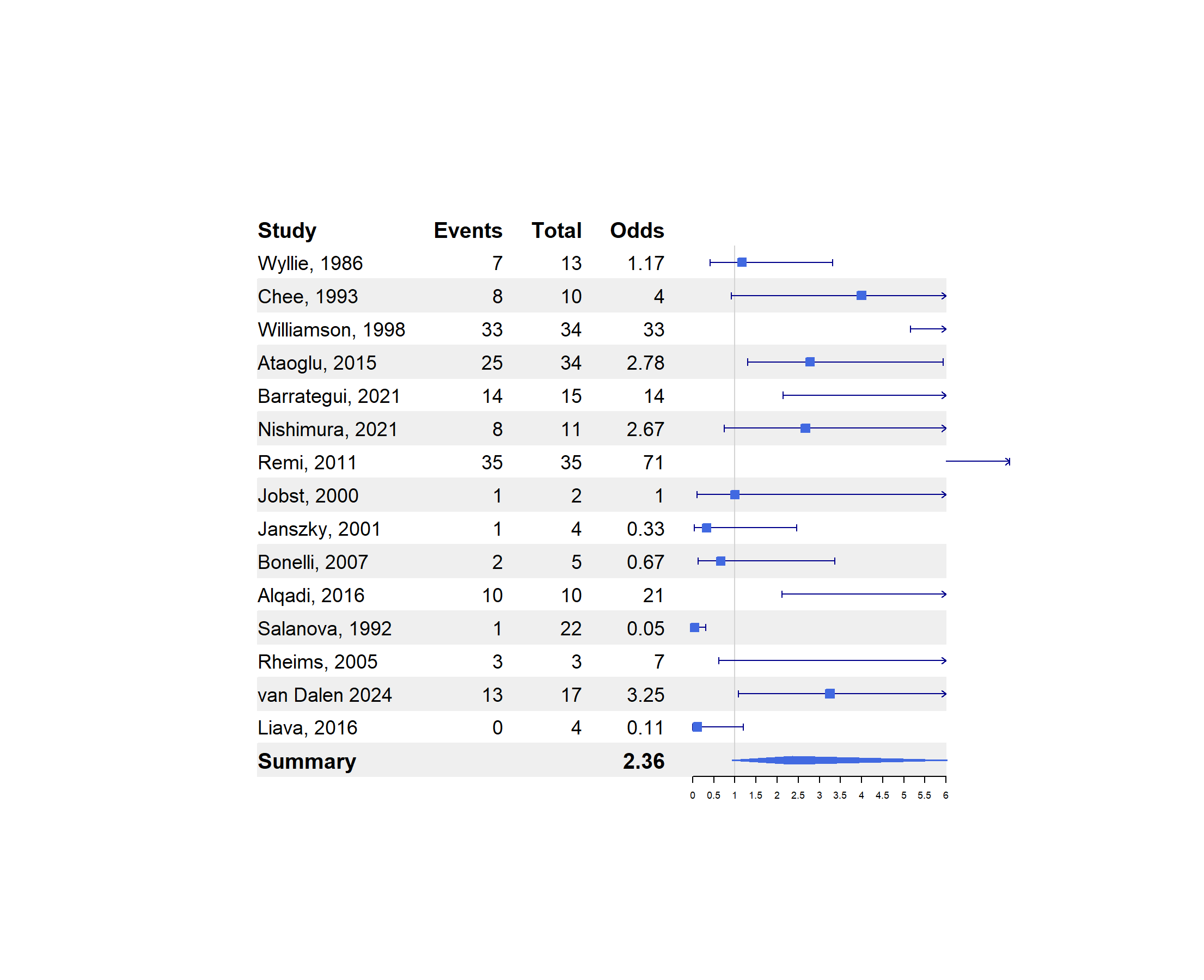

Supplement: Supplementary file 1 — Figure S1. [file EPD2-27-568-s001.tiff]

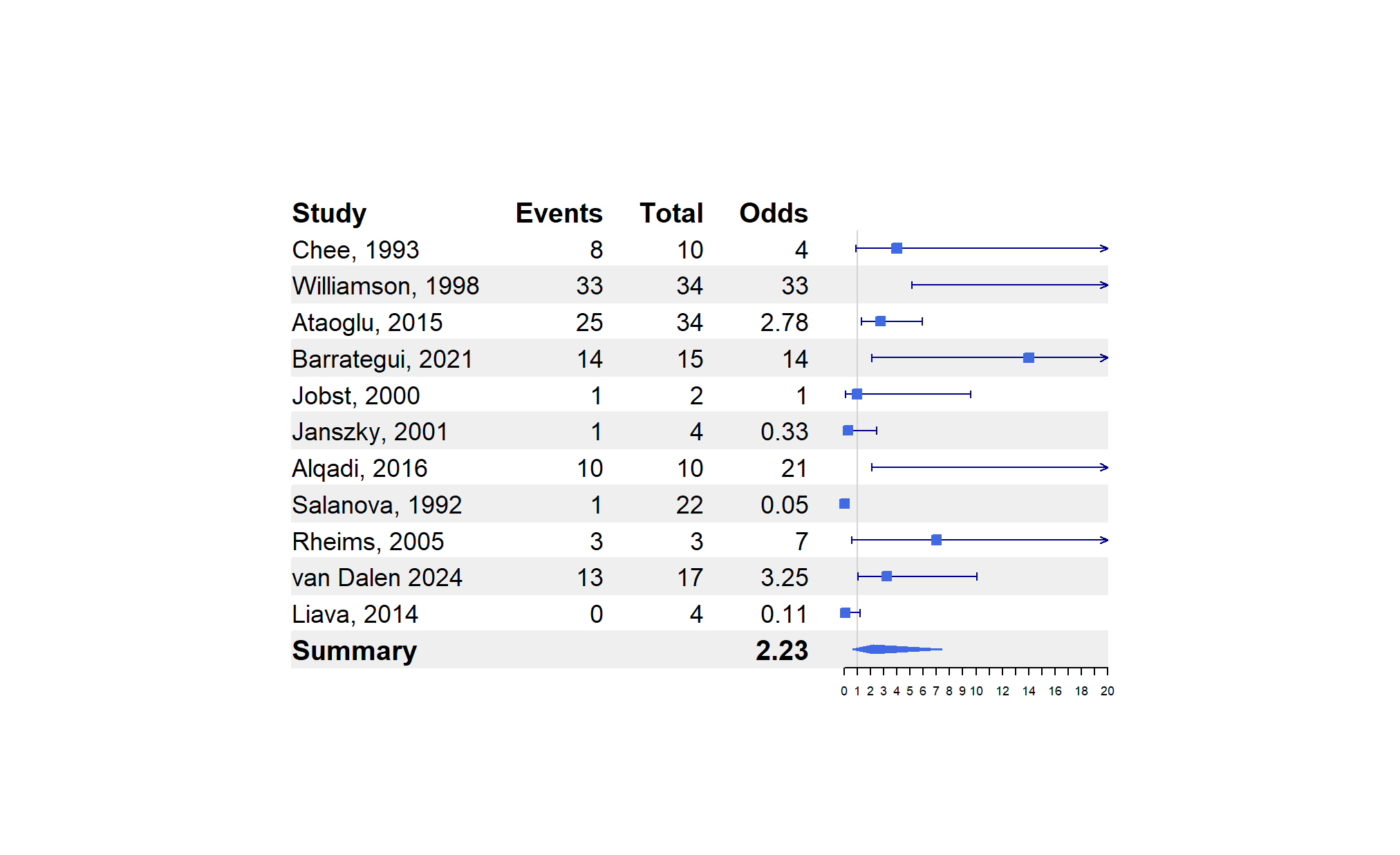

Supplement: Supplementary file 2 — Figure S2. [file EPD2-27-568-s004.tiff]

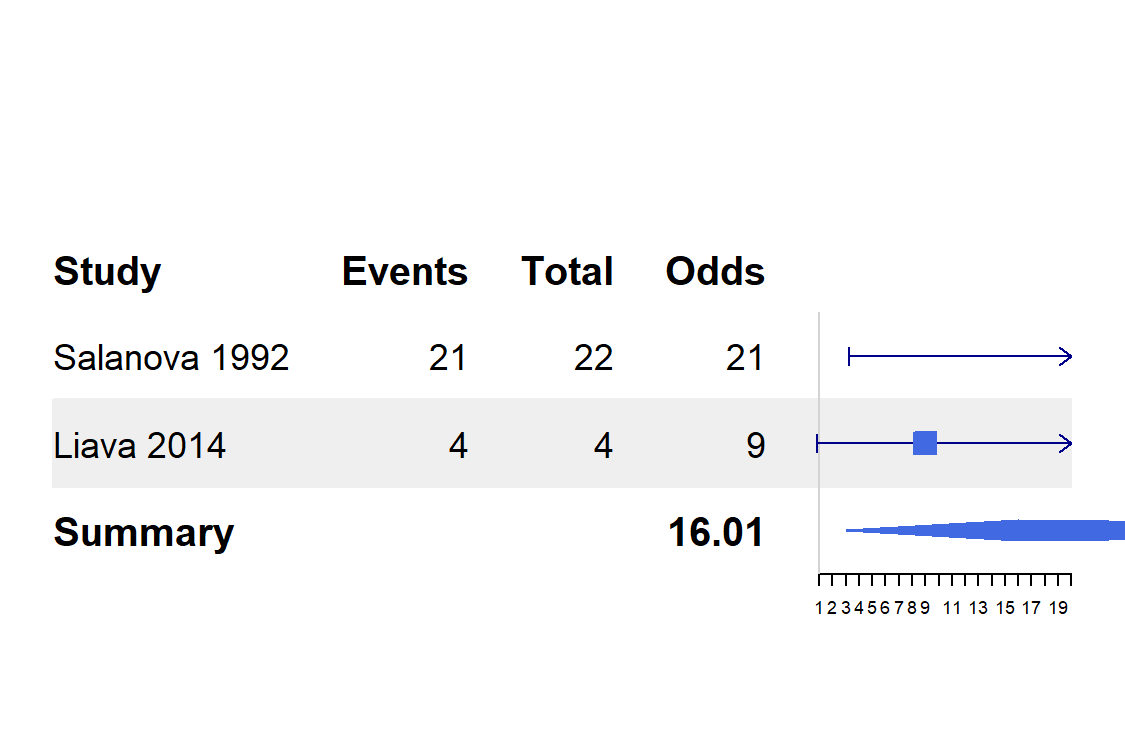

Supplement: Supplementary file 3 — Figure S3. [file EPD2-27-568-s008.tiff]

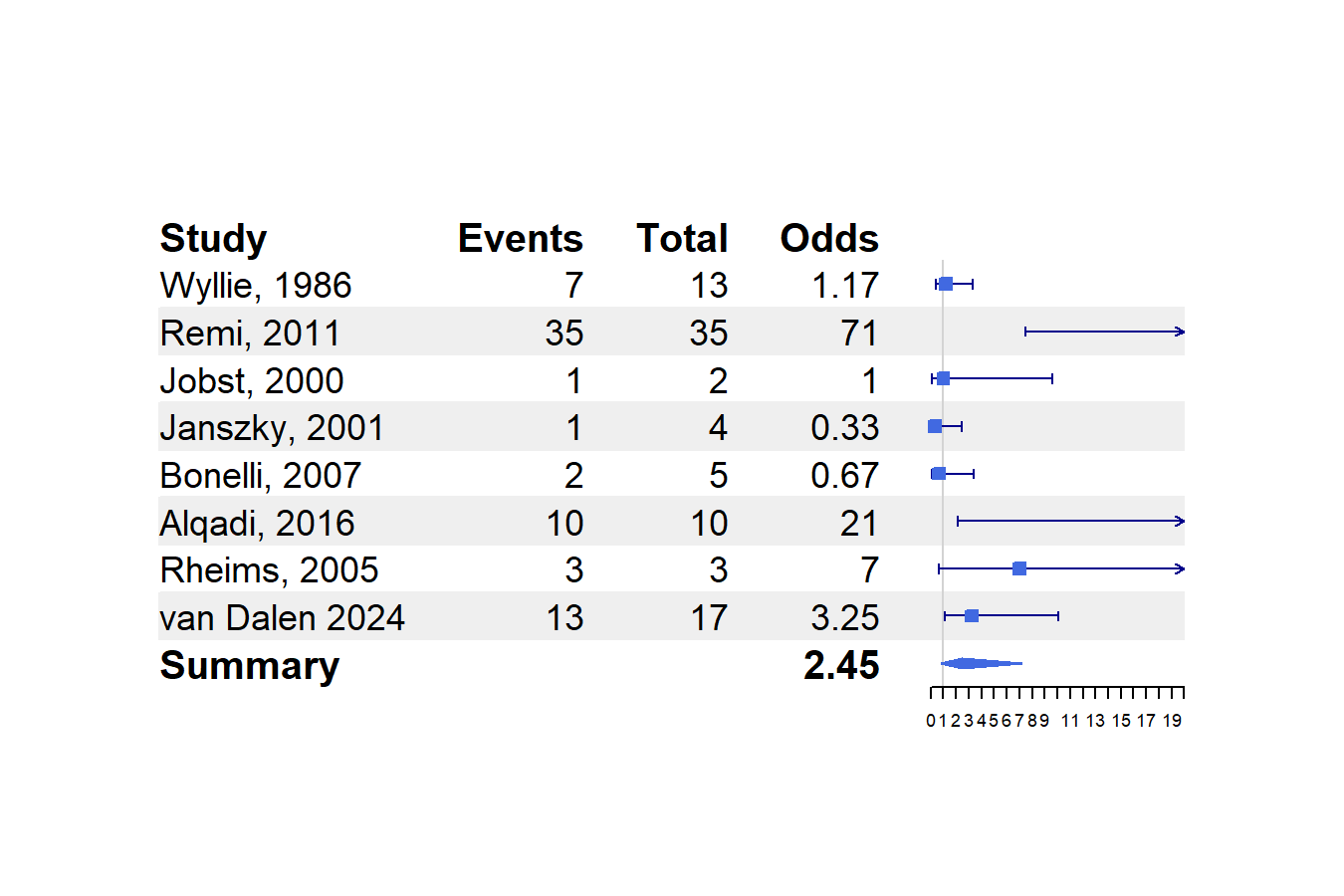

Supplement: Supplementary file 4 — Figure S4. [file EPD2-27-568-s003.tiff]

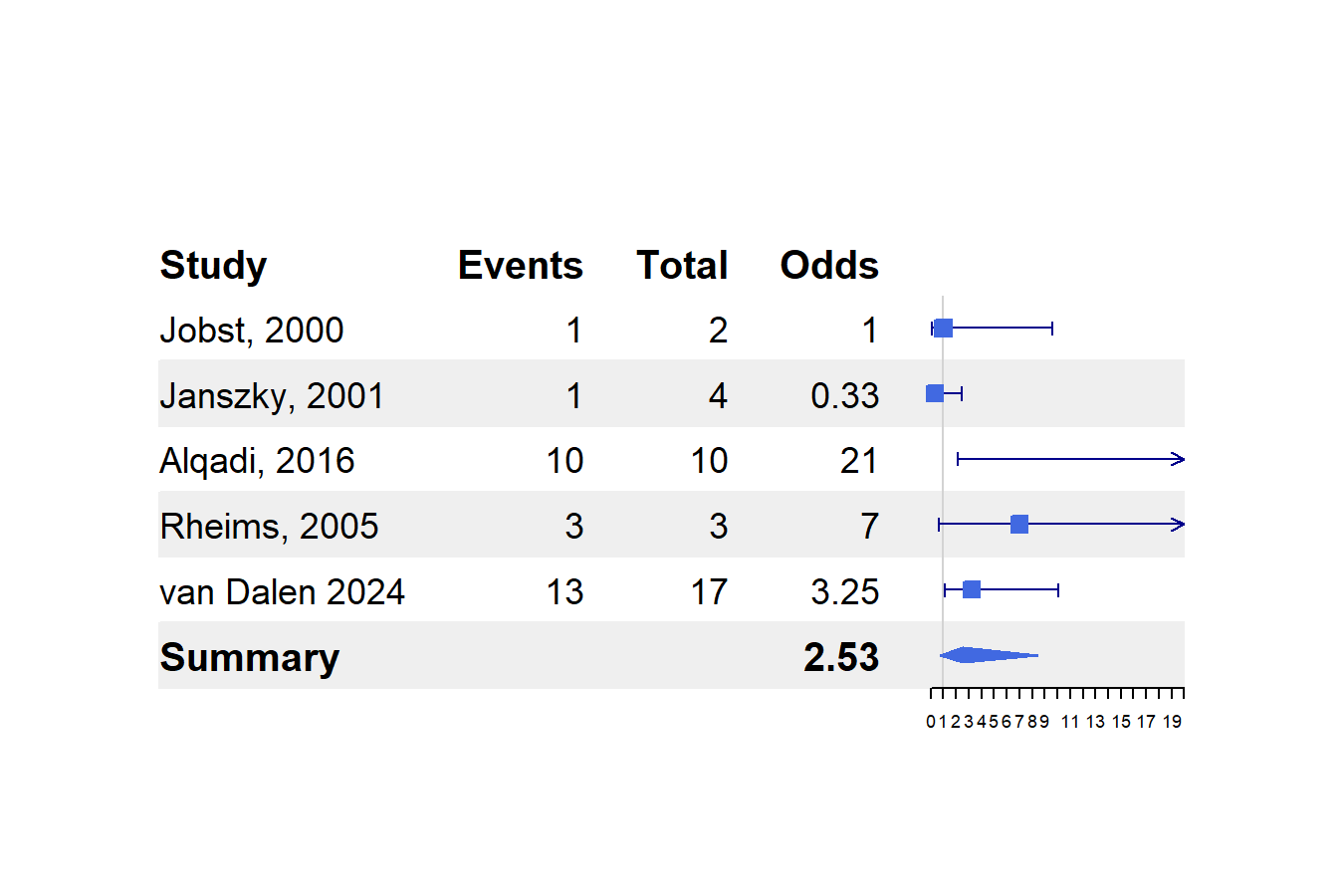

Supplement: Supplementary file 5 — Figure S5. [file EPD2-27-568-s002.tiff]
