# Supplementary material for: Lateralizing value of ictal head turning: A systematic review and meta‐analysis
Source: Epileptic Disord. 2025 May 23;27(4):568–78. doi: 10.1002/epd2.70046 (PMC12398197; doi:10.1002/epd2.70046)
Supplement: Supplementary file 6 — Table S1. [file EPD2-27-568-s005.docx]

Supplementary table2. The method of localization of the epileptogenic zone in the papers included in the analysis of non-versive head turning

| **Study** | **Author, year** | **Surgical outcome (Engel score)** | **Video EEG or videotape analysis** | **Subdural electrodes** | **sEEG** |
| --- | --- | --- | --- | --- | --- |
| 1 | Wyllie, 1986 [3] |  | video EEG | subdural grids 8/37 |  |
| 2 | Chee, 1993 [14] | surgical outcome | videotape analysis |  |  |
| 3 | Williamson, 1998 [20] | surgical outcome | videotape analysis, long-term scalp EEG |  | sEEG |
| 4 | Ataoğlu, 2015 [21] | sugical outcome | video EEG |  |  |
| 5 | Abarrategui, 2021 [25] | surgical outcome | video EEG |  | sEEG 17.4% |
| 6 | Nishimura, 2021 [46] | surgical outcome |  |  | sEEG 100% |
| 7 | Rémi, 2011 [28] | surgical outcome + not operated pts | surface EEG + quantitative video analysis |  |  |
| 8 | Jobst, 2000 [30] | surgical outcome | video EEG, videotape analysis | subdural electrodes | depth electrodes |
| 9 | Janszky, 2001 [31] | surgical outcome | video EEG | subdural electrodes |  |
| 10 | Bonelli, 2007 [32] | surgical outcome | video EEG | subdural eledtrodes |  |
| 11 | Alqadi, 2016 [47] | surgical outcome | video | subdural grids |  |
| 12 | Salanova, 1992 [42] | surgical outocome | video EEG | electrocorticogaphy | sEEG 6 |
| 13 | Rheims, 2005 [41] | surgical outcome | video EEG |  | sEEG |
| 14 | van Dalen 2024 [43] | surgical outcome | video EEG |  |  |
| 15 | Liava, 2014 [48] | surgical outcome | video EEG |  | sEEG |
