# Supplementary material for: Lateralizing value of ictal head turning: A systematic review and meta‐analysis
Source: Epileptic Disord. 2025 May 23;27(4):568–78. doi: 10.1002/epd2.70046 (PMC12398197; doi:10.1002/epd2.70046)
Supplement: Supplementary file 7 — Table S2. [file EPD2-27-568-s006.docx]

Supplementary table 1. The method of localization of the epileptogenic zone in the papers included in the analysis of versive head turning

| **Study** | **Author, year** | **Surgical outcome (Engel score)** | **Video EEG or videotape analysis** | **Subdural electrodes** | **sEEG** |
| --- | --- | --- | --- | --- | --- |
| 1 | Elwan, 2018 [10] | surgical outcome | video EEG | subdural grids | sEEG |
| 2 | Fotedar, 2022 [11] |  | video EEG |  |  |
| 3 | Wyllie, 1986 [3] |  | video EEG | subdural grids |  |
| 4 | Bleasel 1997 [12] | surgical outcome | videotape analysis |  |  |
| 5 | Jayakar, 1992 [13] | sugical outcome | video EEG | subdural EEG | depth EEG |
| 6 | Chee, 1993 [14] | surgical outcome | videotape analysis |  |  |
| 7 | Ochs, 1984 [15] |  | video EEG |  | depth electordes 42% of seizures |
| 8 | Kotagal, 1989 [16] | surgical outcome | videotape analysis | subdural grids and strips 10 patients |  |
| 9 | Shukla, 2002 [17] |  | video EEG |  |  |
| 10 | Kotagal, 2000 [18] | surgical outcome | videotape analysis |  |  |
| 11 | Marks, 1998 [19] | surgical outcome | videotape analysis |  |  |
| 12 | Williamson, 1998 [20] | surgical outcome | videotape analysis, long-term scalp EEG |  | sEEG |
| 13 | Ataoğlu, 2015 [21] | sugical outcome | video EEG |  |  |
| 14 | Wang, 2020 [22] | surgical outcome | video EEG |  | sEEG (38%) |
| 15 | Wang, 2013 [23] | surgical outcome | video EEG |  |  |
| 16 | Duchowny, 1994 [24] | surgical outcome |  | subdural | sEEG |
| 17 | Abarrategui, 2021 [25] | surgical outcome | video EEG |  | sEEG 17.4% |
| 18 | Martinez-Lizana, 2022 [26] | surgical outcome | video EEG |  | sEEG 21% |
| 19 | Wyllie, 1986 [27] |  | videotape analysis |  |  |
| 20 | Rémi, 2011 [28] | surgical outcome + not operated pts | surface EEG + quantitative video analysis |  |  |
| 21 | Salanova, 1995 [29] | surgical outcome | video EEG | subdural electordes |  |
| 22 | Jobst, 2000 [30] | surgical outcome | video EEG, videotape analysis | subdural electrodes | depth electrodes |
| 23 | Janszky, 2001 [31] | surgical outcome | video EEG | subdural electrodes |  |
| 24 | Bonelli, 2007 [32] | surgical outcome | video EEG | subdural grids |  |
| 25 | Chou, 2020 [33] | surgical outcome | video EEG | subdural grids or strips 5 pts | sEEG (9 patients) |
| 26 | Morris, 1988 [34] |  | videotape analysis (video EEG?) | subdural electrodes |  |
| 27 | Lee, 2008 [35] | sugical outcome | video EEG | subdural electrodes 76% |  |
| 28 | Harvey, 1993 [36] | surical outcome | video EEG |  |  |
| 29 | Usui, 2011 [37] | surgical outcome | video EEG |  |  |
| 30 | Boesebeck, 2002 [38] | surgical outcome | video EEG | subdural grids | sEEG |
| 31 | Olbrich, 2002 [39] | surgical outcome | video EEG | subdural grids |  |
| 32 | Fernandez, 2018 [40] |  | video EEG |  |  |
| 33 | Rheims, 2005 [41] | surgical outcome | video EEG |  | sEEG |
| 34 | Salanova, 1992 [42] | surgical outcome | video EEG | electrocorticography | sEEG 6 |
| 35 | van Dalen, 2024 [43] | surgical outcome | video EEG |  |  |
| 36 | Bartolomei, 2011 [44] | surgical outcome 10/17 pts | video EEG |  | sEEG 100% |
| 37 | Yang, 2018 [45] | surgical outcome | video EEG |  | sEEG 100% |
