# Supplementary material for: Lateralizing value of ictal head turning: A systematic review and meta‐analysis
Source: Epileptic Disord. 2025 May 23;27(4):568–78. doi: 10.1002/epd2.70046 (PMC12398197; doi:10.1002/epd2.70046)
Supplement: Supplementary file 8 — Figure Caption. [file EPD2-27-568-s007.docx]

**Supplementary figure 1.**

**Forrest plot for the ipsilateral localization of the epileptogenic zone in seizures with non-versive head turning – all included studies**

**Supplementary Figure 2.**

**Forrest plot for the ipsilateral localization of the epileptogenic zone in seizures with non-versive head turning – high-quality studies**

**Supplementary Figure 3.**

**Forrest plot for the contralateral localization of the epileptogenic zone in seizures with non-versive head turning in occipital cases**

**Supplementary figure 4.**

**Forrest plot for the ipsilateral localization of the epileptogenic zone in seizures with non-versive head turning in frontal cases – all included studies**

**Supplementary Figure 5.**

**Forrest plot for the ipsilateral localization of the epileptogenic zone in seizures with non-versive head turning in frontal cases – high-quality studies**
